# Ingested Australasian Snapper (Chrysophrys auratus) Bone Migration to C5 Vertebral Body

**DOI:** 10.7759/cureus.56301

**Published:** 2024-03-16

**Authors:** Fadzrul Abbas Mohamed Ramlee, Nurulizzah Ibrahim, Muhammad H Hashim

**Affiliations:** 1 Orthopaedic Surgery, Hospital Pengajar University Putra Malaysia, Serdang, MYS; 2 Orthopaedics and Traumatology, Sultan Idris Shah Hospital, Serdang, MYS

**Keywords:** odynophagia, neck exploration, deep neck abscess, fish bone perforation, fish bone ingestion

## Abstract

Fishbone ingestion has been reported multiple times previously as a cause of oesophageal perforation. This is a surgical emergency that needs to be identified early to ensure immediate medical attention. This report presents the case of a 70-year-old patient with laryngeal perforation and the migration of a *Chrysophrys auratus* (Australasian snapper) fishbone to the C5 vertebral body. It is hypothesized that the fishbone migrated from the larynx to the visceral fascia and prevertebral fascia before lodging in between the intramuscular substance of the longus coli muscle. Multiple imaging modalities were used to identify and locate the foreign body, including flexible nasopharyngoscopy, esophagogastroduodenoscopy, and a computed tomography (CT) scan of the neck. The exploration of the neck was done by the ENT team and the orthopaedic spine team via the left anterior cervical approach at the level of the C5 and C6 vertebral bodies. The foreign body was identified (15 mm fishbone) at the left lateral of the C5 body, lodged between the intramuscular substance of the longus coli muscle, and was successfully removed.

## Introduction

Oesophageal perforation is a rare, severe, and challenging surgical emergency that can be caused either iatrogenically, such as through intubation, surgical procedures, or endoscopic examinations, or by non-iatrogenic causes like penetrating trauma or foreign body ingestion [1]. It is associated with a high mortality rate of 40-60% when the treatment is delayed for more than 24 hours [2].

Fishbone ingestion has been reported to be the most common cause of oesophageal perforation. This is a surgical emergency and is associated with life-threatening conditions such as pneumomediastinum, mediastinitis, carotid artery pseudoaneurysm, pleural empyema, septic shock, and death [3]. Most cases presented within the first 24 hours, while others presented with complications following a delayed diagnosis. This report presents the case of a patient with laryngeal perforation and the migration of a fishbone to the C5 vertebral body. 

## Case presentation

**Figure 1:**
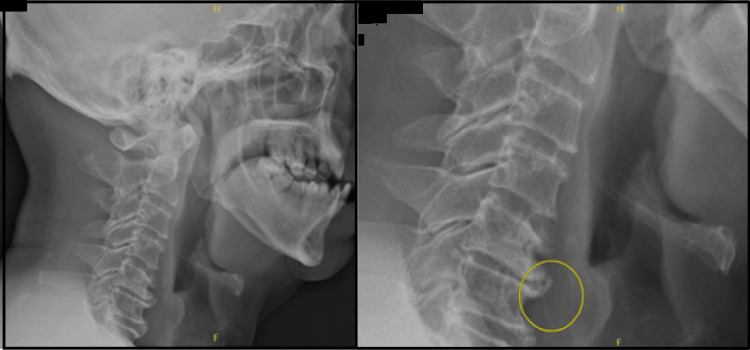
Cervical x-ray of the patient in lateral view showing foreign body in the marked area

A 70-year-old male presented with a three-day history of left throat pain and odynophagia, which is pain on swallowing, following the ingestion of a fishbone from an Australasian Snapper fish (*Chrysophrys auratus*). He was referred to the ENT team after the district hospital found no foreign body on examination. At clinical presentation, there was no swelling and there were no nodes on the neck, but the left throat was tender. Flexible nasopharyngoscopy did not identify the foreign body within the alimentary canal. Esophagogastroduodenoscopy examinations also failed to identify the foreign body and findings suggestive of antral gastritis. The ENT team proceeded with an examination under anesthesia and a direct laryngoscopy of the oropharynx and neck. Intraoperatively, they noted a small entry point wound with no ulcer or bleeding over the posterolateral wall of the left pyriform fossa. A cervical x-ray was also done; Figure 1 shows the cervical x-ray of the patient in lateral view with a radiopaque foreign body highlighted in the marked area near C5/C6. There is no increase in prevertebral soft tissue shadow with degenerative cervical spine bony changes.

**Figure 2:**
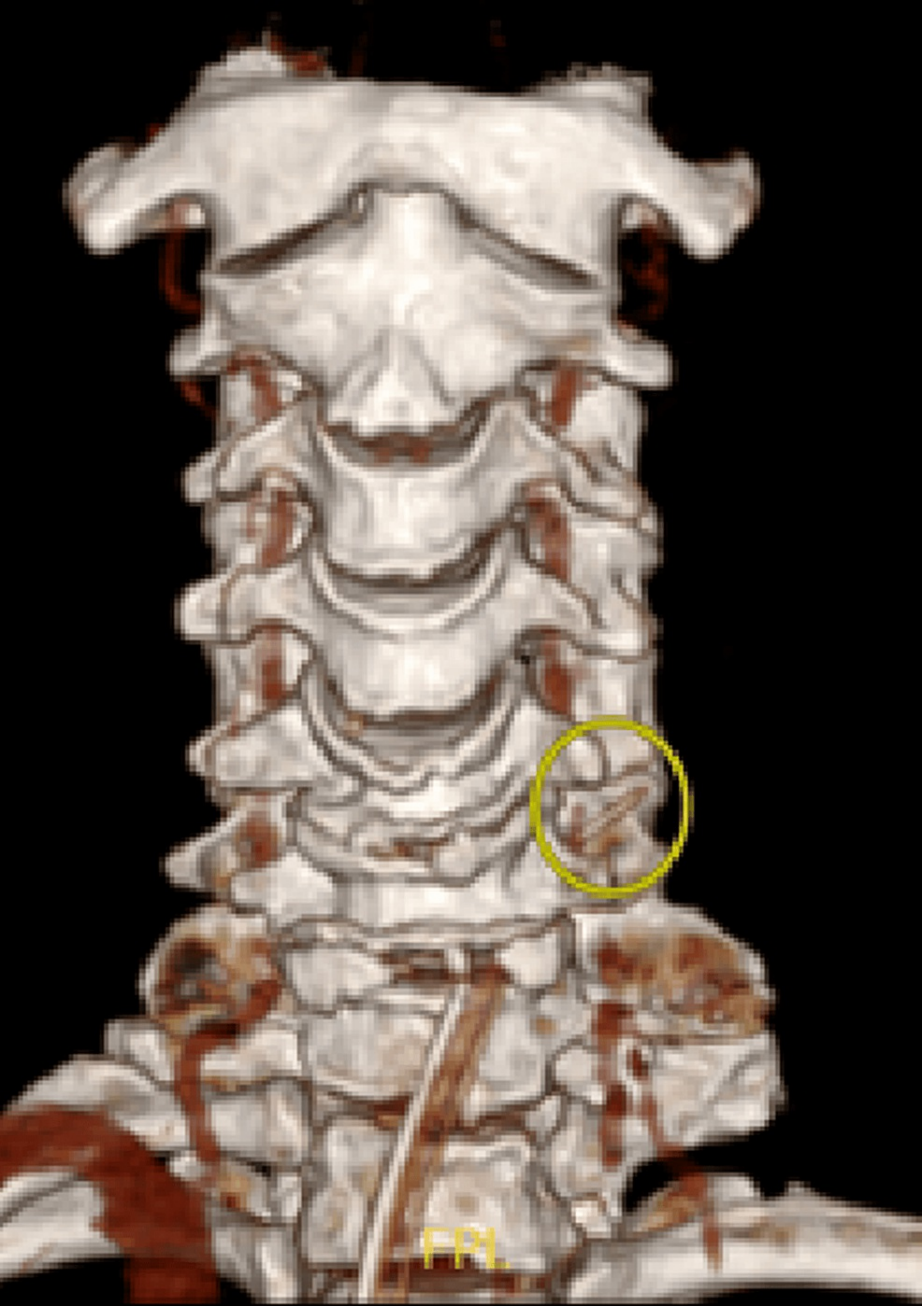
CT cervical (coronal view) of the patient in three-dimensional reconstruction showing the foreign body in the marked area

Subsequently, the patient was subjected to a CT neck scan with 3D reconstruction. This revealed a linear hyperdense structure embedded at the pharyngeal wall posterior to the left cricoid, likely to represent an embedded foreign body associated with prevertebral collection which was seen from C3 to C6. There was also no evidence of mediastinitis or oesophageal perforation. Figure 2 shows the cervical CT scan of the patient in three-dimensional (3D) reconstruction, showing the foreign body in the marked area.

Subsequently, the ENT team proceeded with neck exploration and dissection between the left thyroid lamina and the carotid sheath, the posterior border of the sternoclavicomastoid muscle, and the carotid sheath, but the foreign body was not identified at the predetermined location based on radiological imaging.

**Figure 3:**
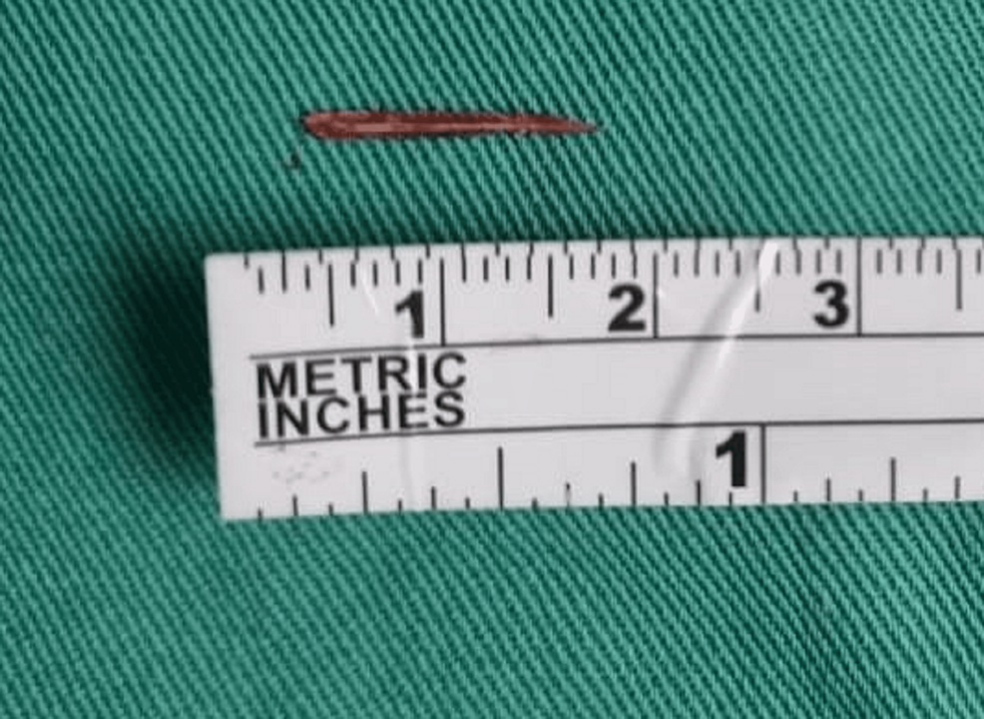
Extracted foreign body (serrated fishbone), measuring 15 mm

In view of suspecting migration of the foreign body, a repeat CT scan was done and the foreign body was located 0.94 cm from the left posterior lateral wall traversing posterior obliquely from the ryles tube. It was seen within the lateral part of the left longus colli muscle. Re-exploration was done by the ENT team with the orthopaedic spine team via the left anterior cervical approach at the level of the C5/C6 vertebral bodies. The foreign body was eventually identified (15 mm serrated fishbone) at the left lateral of the C5 body, lodged between the intramuscular substance of the longus coli muscle, and was successfully removed. Figure 3 shows an intraoperative image of the foreign body (serrated fishbone) extracted from the patient, measuring 15 mm.

Postoperatively, the patient desaturated post extubation and failed to maintain adequate oxygenation using non-invasive ventilation support. Therefore, he was re-intubated prior to the re-exploration procedure. Postoperatively, he required inotropic support with a high ventilation setting to maintain oxygenation. His chest x-ray showed worsening haziness with acute respiratory distress syndrome (ARDS) changes. He succumbed to death on postoperative day 6 from septic shock secondary to pneumonia.

When evaluating the location of the foreign body in close proximity to the C5 vertebral body, the surgeons came to an agreement that it was necessary to perform extensive explorations as it may dislodged further into the spinal canal and subsequently complicated with epidural abscess, dura tear, or even risk of patient developing neurological deficit. However, in the event that it was left in situ, there is a risk of developing an abscess and further migration into surrounding structures, which could cause pseudoaneurysm.

## Discussion

In our case, it is believed that the fishbone migrated from the larynx to the visceral fascia and prevertebral fascia before lodging in between the intramuscular substance of the longus coli muscle. As the fishbone was serrated, it had unidirectional movement following oesophageal peristalsis, neck movement, and manipulation. If a migrated fishbone is left untreated, it may cause pneumomediastinum, mediastinitis, carotid artery pseudoaneurysm, pleural empyema, septic shock, and death, as mentioned previously [1]. In the literature, other complications related to fishbone ingestion have been reported, including vocal cord paresis, hepatic abscess, aortic-oesophageal fistula, subclavian oesophageal fistula, carotid rupture, subclavian artery pseudoaneurysm, aortic pseudoaneurysm, false oesophageal hiatus hernia, and pharyngeal perforation [4]. In 2020, Lambert et al. reported a case of tilapia fishbone ingestion causing oesophageal perforation and neck abscess, which was managed by right cervicotomy [2]. In the current case, there were a few complications worth discussing. Firstly, the preoperative complications following ingestion of the fishbone. The patient developed prevertebral collection extending from C3 to C6 which most likely represents abscess formation. Following the open exploration of the neck, the patient suffered from complications of general anaesthesia where he desaturates post extubation due to lung complications.

Approximately 50% of patients affected by fishbone ingestion are misdiagnosed initially at the time of presentation [4]. For example, in 2016 Chen et al. reported a case of thoracic spinal epidural abscess caused by fishbone penetration following delayed diagnosis and initial treatment [4]. Another case report from 2010 narrated that a patient presented with a spinal epidural abscess as a complication of a delayed diagnosis of fishbone ingestion [5]. Therefore, thorough history-taking and clinical examination with the supplementation of imaging modalities are vital in making the diagnosis and initiating early treatment. A plain radiograph has been reported to only have 39% sensitivity and a specificity of 72% [6]. On the other hand, multidirectional CT has a sensitivity of 90-100% and a specificity of 93.7-100% in detecting foreign bodies [7]. Good imaging may guide surgeons to evaluate the foreign body and the surgical approach for exploration. Indications for surgical exploration should include migration of foreign bodies, formation of abscess, and perforation of surrounding structures. Successful removal of foreign bodies may prevent catastrophic and debilitating complications as mentioned previously.

## Conclusions

Ingestion of fishbones commonly causes serious complications. Early diagnosis is of utmost importance to initiate treatment and surgical removal. There are a few lessons that can be learned, and improved upon, from this case. Following the patient's history, clinical imaging is of vital importance to identify and locate the foreign body before subjecting the patient to invasive procedures such as scopes. Secondly, a multidisciplinary approach should be undertaken to manage such a patient which includes ENT, gastroenterology, and orthopaedics teams before embarking on surgical exploration. 
